# Mucosal Taï Forest virus infection causes disease in ferrets

**DOI:** 10.1371/journal.ppat.1013579

**Published:** 2025-10-13

**Authors:** Paige Fletcher, Kyle L. O’Donnell, Joseph F. Rhoderick, Corey W. Henderson, Atsushi Okumura, Trenton Bushmaker, Kathleen Cordova, Greg Saturday, Andrea Marzi

**Affiliations:** 1 Laboratory of Virology, Division of Intramural Research, National Institute of Allergy and Infectious Diseases, National Institutes of Health, Hamilton, Montana, United States of America; 2 Rocky Mountain Veterinary Branch, Division of Intramural Research, National Institute of Allergy and Infectious Diseases, National Institutes of Health, Hamilton, Montana, United States of America; Icahn School of Medicine at Mount Sinai, UNITED STATES OF AMERICA

## Abstract

The filovirus Taï Forest virus (TAFV) caused a single human case of infection originating from a chimpanzee outbreak, demonstrating that humans are susceptible to TAFV infection. Existing animal disease models use intramuscular (IM) infection; however, natural filovirus infection likely occurs mucosal. We aimed to develop a ferret disease model by inoculation of TAFV by the IM, intranasal (IN), or aerosol routes. The IM group showed minimal signs of disease while IN and aerosol inoculations resulted in moderate to severe disease and partial lethality. The surviving IN or IM TAFV-infected ferrets were rechallenged IM or IN with Ebola virus (EBOV) as a pilot study assessing the cross-protection potential between these closely related viruses. Only ferrets IN-inoculated with TAFV and IN-inoculated with EBOV were protected from disease, all others succumbed to disease after EBOV infection. This data shows that ferrets are a feasible model to assess TAFV pathogenicity by mucosal exposure routes and that possible cross-protection between TAFV and EBOV may be achieved upon mucosal exposure.

## Author summary

Filoviruses are zoonotic pathogens known to cause outbreaks of hemorrhagic disease in humans. Filovirus human-to-human transmission occurs via bodily fluids likely by exposure of mucosal surfaces. Taï Forest virus (TAFV) is a lesser-known human pathogenic filovirus. There are no well-established small animal disease models for TAFV, specifically no natural TAFV exposure models. We aimed to fill this knowledge gap and developed a small animal disease model using a mucosal route of infection.

Ferrets are an established model of mucosal infection for many viruses, such as influenza, and have been shown to be susceptible to wildtype filovirus infection. Our study demonstrated that only ferrets infected via the intranasal or aerosol routes with TAFV developed mild to severe disease with partial lethality. The intramuscular group survived without developing disease. A subset of the surviving TAFV-infected ferrets was rechallenged in a pilot study with Ebola virus (EBOV) to assess the cross-protection potential between these closely related filoviruses. Interestingly, only ferrets infected intranasally with TAFV and rechallenged intranasally with EBOV survived. This highlights the importance of mucosal exposure to assess TAFV pathogenicity and the cross-protection potential between TAFV and EBOV. Furthermore, this model presents a new opportunity for countermeasure evaluation against TAFV in a small animal.

## Introduction

Filoviruses are negative-sense, single-stranded RNA viruses classified as select agents, NIAID category A priority agents, and CDC category A bioterrorism agents [1]. Filoviruses are known to cause hemorrhagic disease outbreaks with transmission from human-to-human by bodily fluids which can severely impact public health, requiring special action for public health preparedness [2]. Taï Forest virus (TAFV) is a lesser-known member of the *Filoviridae* family first identified in the Parc National de Taï of Côte d’Ivoire (formerly known as *Ivory Coast ebolavirus*) [3]. To date, it has caused hemorrhagic disease in a single human case originating from a TAFV-infected chimpanzee, demonstrating the human pathogenic potential [4]. There are currently no well-established small animal disease models for TAFV even though it is classified as a category A agent. Recent studies demonstrated uniformly lethal disease in cynomolgus macaques after intramuscular (IM) infection with a low-passage TAFV stock [5]. However, natural filovirus infection likely occurs via mucosal exposure, such as oral, nasal, conjunctival, or vaginal [6,7]. Therefore, we aimed to develop a small animal disease model using a mucosal route of infection.

Ferrets have been shown to develop signs of disease, including death, after wildtype filovirus infection by different exposure routes, including mucosal exposure [8–10]. A previous study reported minimal disease and 100% survival after TAFV infection in ferrets [11]. However, we hypothesized that our low-passage TAFV stock would result in a different disease phenotype in ferrets since a previous study comparing two different TAFV stocks in cynomolgus macaques demonstrated opposing disease phenotypes, where one stock caused minimal disease while this low-passage stock caused severe disease and lethality [5]. These pathogenic differences are most likely due to the genomic differences related to viral editing and replication [5]. This low-passage TAFV stock has a guanine in the editing site of normally 7 uracils in the TAFV glycoprotein (GP) resulting in a amino acid change. It also encodes mutations in viral protein 30 (VP30) and the RNA-dependent RNA polymerase “large” protein (L) which contribute to viral replication [5]. To assess the impacts of pathogenicity based on the exposure route of this same low-passage TAFV stock, ferrets were inoculated by IM, intranasal (IN), or aerosol exposure routes. We found that ferrets exposed via mucosal routes (IN or aerosol) to TAFV developed mild to severe disease.

There are six known viruses in the orthoebolavirus genus with Ebola virus (EBOV) being one of the most well-known human pathogenic viruses. The genomic identity is highly similar between these viruses, especially between the four viruses that cause Ebola disease (EBOV, TAFV, Sudan virus, and Bundibugyo virus). Interestingly, EBOV and TAFV have high sequence conservation in L, VP24, and VP40 [12]. There are also similarities surrounding the GP furin cleavage site between these two viruses [13]. The current dogma in the field is that there is no to limited cross-protection between filovirus species as demonstrated in countermeasure studies using NHP intramuscular infection models [14–16], however, there is minimal data when assessing mucosal routes. Therefore, some surviving TAFV-infected ferrets from the pathogenicity study were rechallenged either IM or IN in a pilot study with EBOV to assess the cross-protection potential due to their sequence similarities. It was observed that IN-exposed TAFV and IN-exposed EBOV ferrets were the only survivors. Overall, our results demonstrate that ferrets are a feasible model to assess TAFV pathogenicity and that possible cross-protection may be achieved by mucosal exposure routes.

## Results

### Ferrets develop disease after mucosal TAFV inoculation

**Fig 1:**
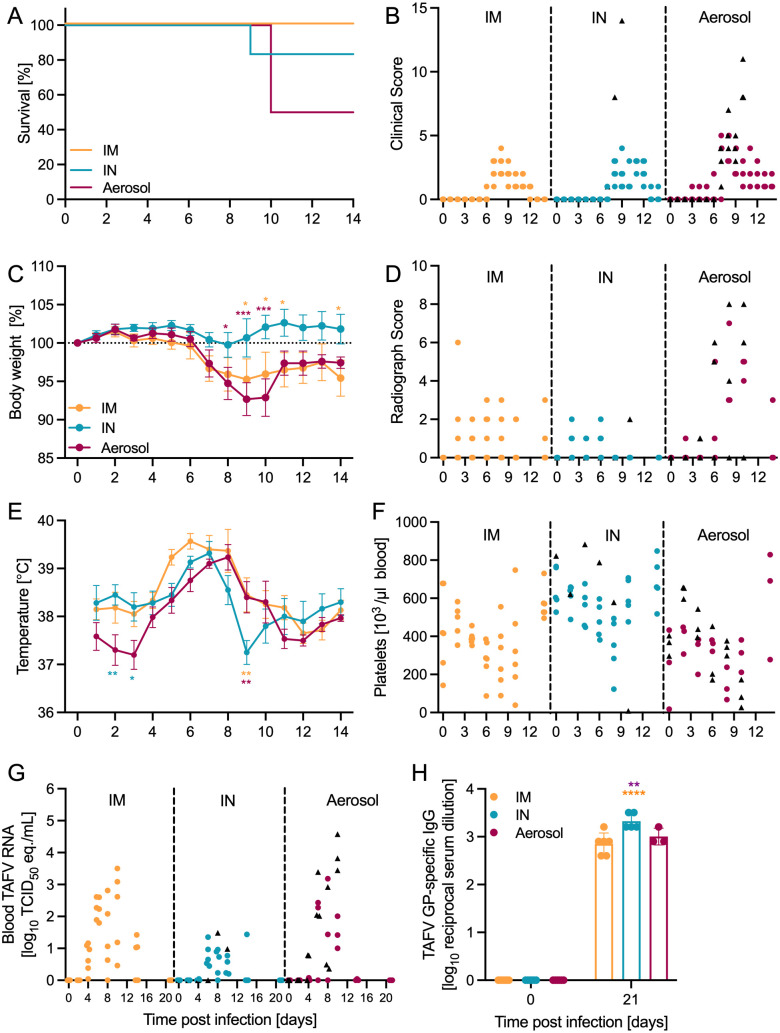
Clinical findings after TAFV exposure in ferrets. Ferrets were inoculated IM, IN, or by aerosol (n = 6/group) with 10,000 TCID_50_ of TAFV. (A) Survival curve, (B) clinical score, (C) body weight percentage normalized to 0 dpi (mean with SEM), (D) lung radiograph score, (E) transponder temperature beginning at 1 dpi depicted as mean with SEM. (F) Platelet counts and (G) TAFV RNA load in whole blood. (H) TAFV GP-specific IgG in serum depicted as geometric mean and geometric standard deviation. ▲Ferrets reaching endpoint criteria. Statistical significance calculated by 2-way ANOVA with Tukey’s multiple comparisons is indicated as *p < 0.05, **p < 0.01, ***p < 0.001, and ****p < 0.0001.

**Fig 2:**
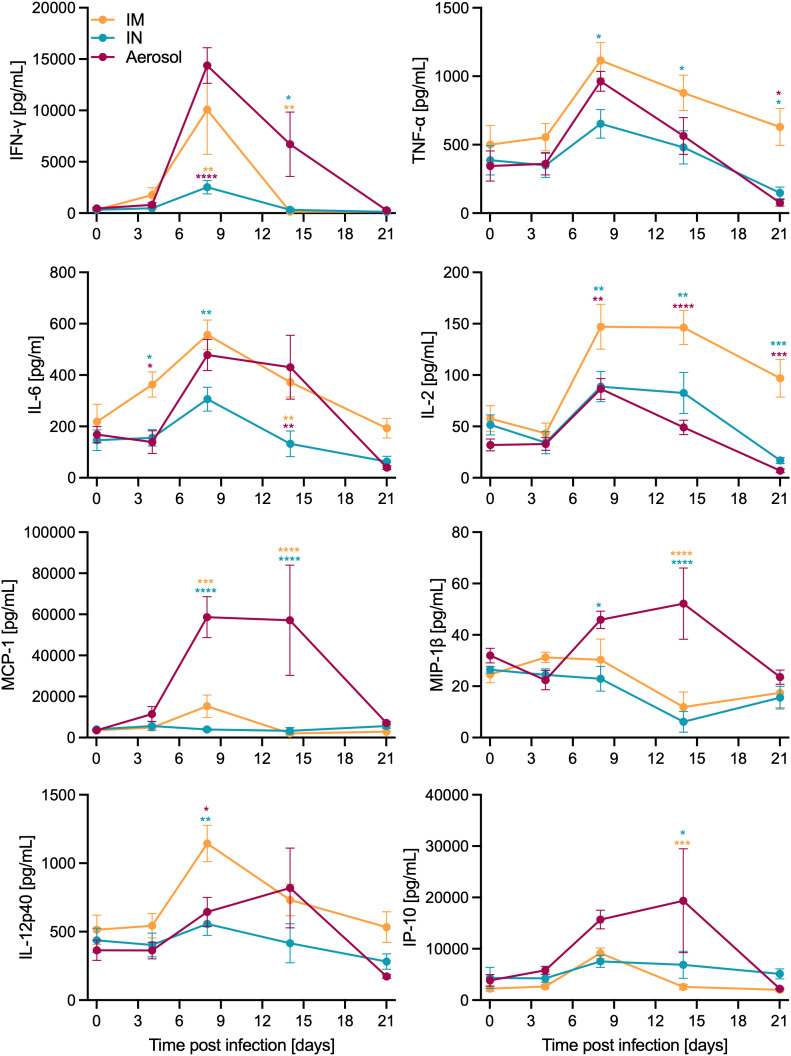
Cytokine and chemokine levels in ferret serum after TAFV exposure. Ferrets were inoculated IM, IN, or by aerosol (n = 6/group) with 10,000 TCID_50_ of TAFV. Levels of select cytokines and chemokines in serum samples collected over time. All depicted as mean with SEM. Statistical significance calculated by 2-way ANOVA with Tukey’s multiple comparisons is indicated as *p < 0.05, **p < 0.01, ***p < 0.001, and ****p < 0.0001.

Ferrets were inoculated with 10,000 median tissue culture infectious doses (TCID_50_) of TAFV by either the IM, IN, or aerosol routes of exposure (n = 6/group). This dose equals ~1,000 plaque-forming units which has been used for several other filovirus ferret infection studies [9]. The IM group demonstrated 100% survival (Fig 1A) with minimal signs of disease. In contrast, mucosal inoculation resulted in 83% (IN) and 50% (aerosol) survival, respectively (Fig 1A). The ferrets meeting endpoint criteria after TAFV infection in the mucosal groups (indicated as black triangles in the figures) reached higher clinical scores (Fig 1B) with hunched posture, ruffled fur, minimal nasal discharge, lethargy, ear changes, orbital tightening, labored breathing, and diarrhea. Weight loss (Fig 1C) and increased radiograph scores (Fig 1D) were also observed in these ferrets. All exposure groups presented increased body temperatures between 6–8 days post-infection (dpi) (Fig 1E) along with lymphocytopenia and neutrophilia on 6 dpi (S1A-S1B Fig). Thrombocytopenia was only observed in the ferrets that reached endpoint criteria in the mucosal groups as indicated as black triangles (Fig 1F). Viremia was detected in all exposure groups starting at 4 dpi with a peak at 8 dpi; virus was cleared from the blood in survivors at the end of the study (21 dpi) (Fig 1G). Normal levels of liver (S1C-S1D Fig) and kidney (S1E-S1F Fig) enzymes were observed between the exposure groups. When assessing the levels of various cytokines and chemokines in the serum, ferrets in the aerosol group had higher IFN-γ, MCP-1, MIP-1β, and IP-10 compared to the other groups (Fig 2). Interestingly, the IM group had increased levels of TNF-α, IL-6, IL-2, and IL-12p40 (Fig 2). Regardless of exposure route, most of these levels peaked around 8 dpi. Viral RNA indicative of TAFV shedding was measured in the throat, nasal, and rectal swabs regardless of exposure group (S2A-S2C Fig). At the time of necropsy, systemic viral spread was observed with the highest levels of TAFV RNA in the ferrets that succumbed to acute disease (S2D Fig). The surviving ferrets seroconverted as indicated by TAFV GP-specific IgG present in the serum at the study end (Fig 1H).

### Histopathologic lesions observed only in mucosal-exposed ferrets

Primary target organs of filovirus disease are the liver and the spleen for which we performed histopathologic analysis. Since ferrets were infected by mucosal routes, the lungs were also assessed. The ferrets meeting endpoint criteria due to acute disease in the IN (1/6) and aerosol (3/6) groups were euthanized 9–10 dpi while some surviving ferrets in the IM (2/6), IN (1/6), and aerosol (3/6) groups were euthanized at study end (21 dpi). The remaining 8 surviving ferrets in the IM and IN groups (n = 4/group) were repurposed for a follow-up cross-protection pilot study.

**Fig 3:**
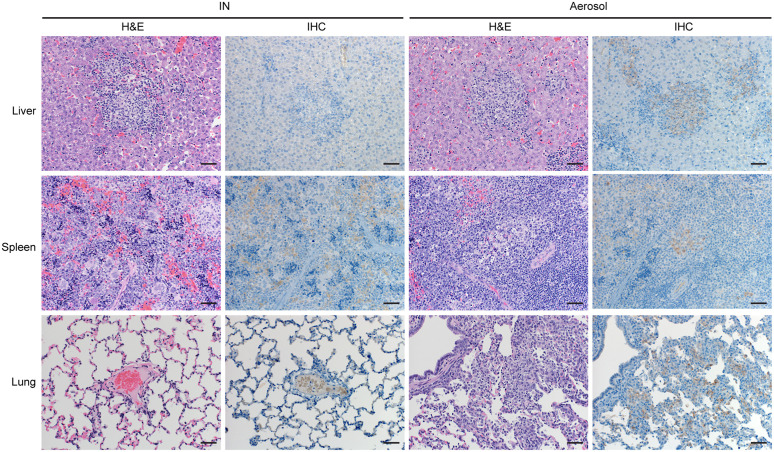
Histopathology of TAFV mucosal-exposed ferrets that succumbed to disease. Ferrets were inoculated IN or by aerosol with 10,000 TCID_50_ of TAFV. Hematoxylin & eosin (H&E) and immunohistochemistry (IHC) staining in tissues of TAFV-exposed ferrets that succumbed at 9-10 dpi. Images 200x magnification. Scale bar represents 50 µm.

The mucosal-infected ferrets that succumbed to acute disease 9–10 dpi demonstrated with pronounced histopathologic changes (Fig 3) compared to the ferrets that survived (S3 Fig). The ferrets that met endpoint criteria showed liver pathology consisting of diffuse microvacuolar change within hepatocytes with multifocal hepatocellular necrosis (Fig 3). There was minimal to moderate neutrophilic inflammation and portal areas had increased lymphohistiocytic infiltrates. The aerosol-exposed ferrets that succumbed to disease on 10 dpi exhibited more pronounced histopathologic changes compared to any other group. Moderate to high viral antigen immunoreactivity within hepatocytes and Kupffer cells was observed in 2/3 aerosol-infected ferrets (Fig 3). Splenic pathology consisted of marked hypercellularity of the red pulp with multifocal areas of necrosis and neutrophilic infiltrates. Moderate to diffuse immunoreactivity was observed in macrophages within the red pulp in 2/3 aerosol-infected ferrets (Fig 3). Pulmonary pathology in the aerosol-infected ferrets displayed moderate to severe interstitial pneumonia, abundant alveolar neutrophilic and histiocytic exudate, moderate to marked perivascular edema with vasculitis and mild to marked bronchiolar exudate. Moderate amounts of viral antigen were present predominately within macrophages, endothelial cells, and perivascular alveolar septa.

In contrast, the surviving ferrets euthanized on 21 dpi had no histopathologic signs of filovirus disease in the liver or the spleen regardless of exposure route (S3 Fig). Lung samples only from the aerosol group (n = 3) exhibited minimal to moderate interstitial pneumonia at 21 dpi (S1 Table).

### Pilot study: Cross-protection between IN-exposed TAFV and EBOV ferrets only

**Fig 4:**
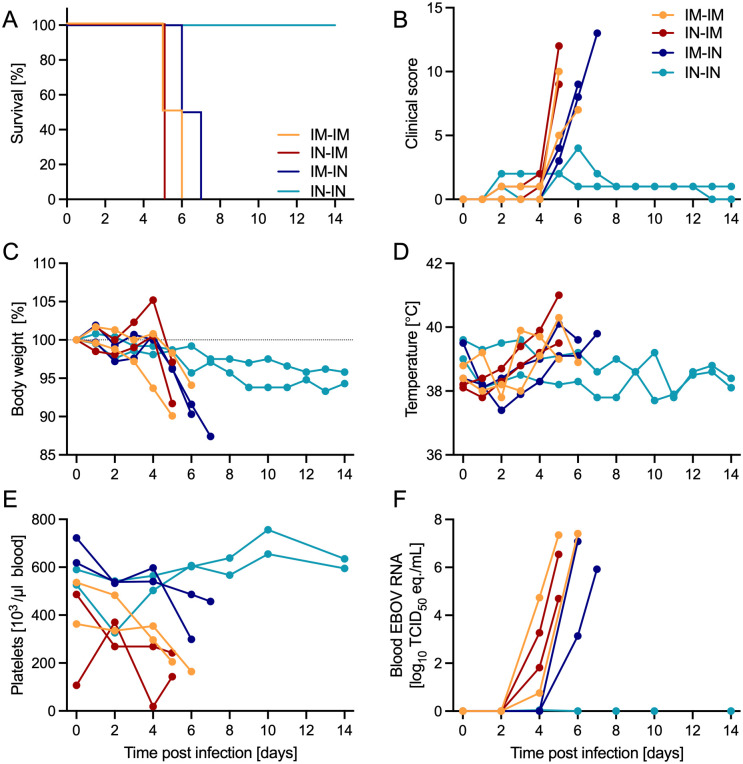
Clinical findings after EBOV rechallenge in ferrets. Ferrets were exposed IM or IN to 1,000 TCID_50_ of EBOV (n = 2/group) after surviving TAFV inoculation. **(A)** Survival curve, (B) clinical score, (C) body weight percentage normalized to 0 dpi, and (D) transponder temperature. **(E)** Platelet counts and **(F)** EBOV RNA in whole blood.

Research on cross-protection between TAFV and other filoviruses is currently limited. Since there was a robust TAFV GP-specific IgG response in the ferret survivors, we decided to assess cross-protection with an EBOV rechallenge pilot study. Surviving IM or IN TAFV-exposed ferrets were inoculated with 1,000 TCID_50_ of EBOV by either IM or IN routes (n = 4/inoculation). The exposure groups (n = 2/group) were as follows: 1) IM-exposed TAFV and IM-exposed EBOV (IM-IM; n = 2 male), 2) IN-exposed TAFV and IM-exposed EBOV (IN-IM; n = 2 female), 3) IM-exposed TAFV and IN-exposed EBOV (IM-IN; n = 2 male), or 4) IN-exposed TAFV and IN-exposed EBOV (IN-IN; n = 2 female). The IN-IN group was the only group to survive both TAFV and EBOV exposures with all other groups succumbing to EBOV disease between 5–7 dpi (Fig 4A). Classical disease was observed in these 6 ferrets as documented by increased clinical scores (Fig 4B) including hunched posture, ruffled fur, lethargy, labored breathing, orbital tightening, ear changes, and diarrhea. Body weight loss (Fig 4C), increased body temperature (Fig 4D), thrombocytopenia (Fig 4E), and viremia (Fig 4F) were also observed in ferrets meeting endpoint criteria. Ferrets that succumbed to disease presented with lymphocytopenia and neutrophilia (S4A-S4B Fig). The IM-IM group presented with higher liver (S4C-S4D Fig) and kidney (S4E-S4F Fig) enzyme levels in the serum compared to the other groups.

EBOV RNA was not detected in any blood samples collected from the IN-IN group throughout the study (Fig 4F). Minimal amounts of viral RNA were detected in swabs of the IN-IN group on 6 dpi compared to the other groups which demonstrated increased viral shedding (S5A-S5C Fig). The IN-IN group had no detectable EBOV RNA in the urine or the tissue samples collected at study end (S5D Fig). In contrast, high amounts of EBOV RNA were measured in samples collected from the ferrets succumbing to acute disease in the IM-IM, IN-IM, and IM-IN groups (S5D Fig). The IN-IN ferrets had normal levels of inflammatory cytokines and chemokines in the serum compared to the other groups with the IM-IM group presenting with the highest levels (S6 Fig).

**Fig 5:**
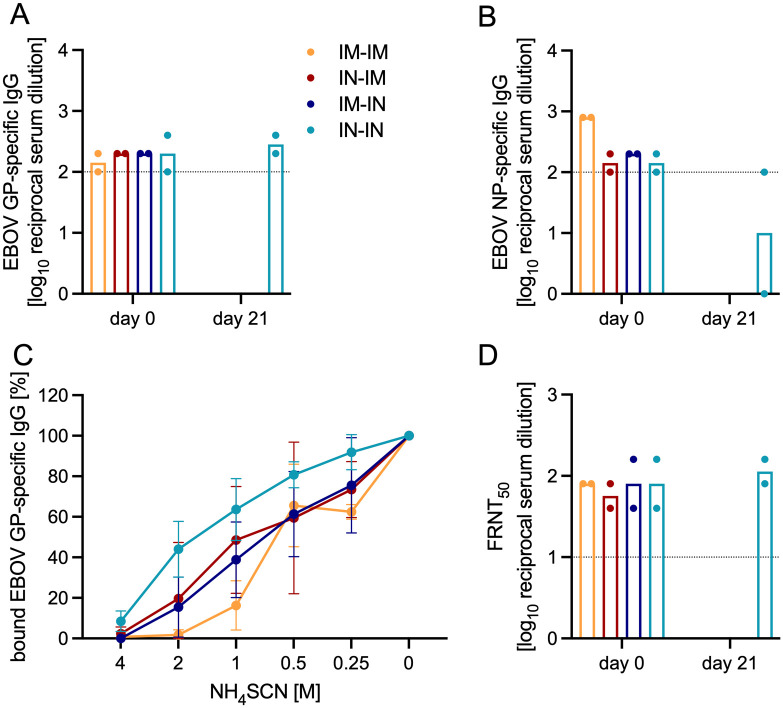
Serology assessment after EBOV rechallenge in ferrets. Ferrets were exposed IM or IN to 1,000 TCID_50_ of EBOV (n = 2/group) after surviving TAFV inoculation. **(A)** EBOV GP- and **(B)** EBOV NP-specific IgG in the serum. **(C)** Percentage of bound EBOV GP-specific IgG shown as mean with SD. **(D)** Neutralization activity presented as 50% reduction of GFP-positive cells (FRNT_50_). (A, B, **D)** Geometric mean is depicted.

To better understand the survival of the IN-exposed TAFV and EBOV ferrets, we assessed the humoral immune response. The EBOV GP-specific IgG response showed similar levels between all groups on 0 dpi and remained constant for the IN-IN survival group on 21 dpi (Fig 5A). The EBOV NP-specific IgG levels were similar between the IN-IM, IM-IN, and IN-IN groups on 0 dpi while the IM-IM group was trending slightly higher (Fig 5B). Interestingly, one ferret in the IN-IN survival group had decreased EBOV NP-specific IgG levels on 21 dpi while the other ferret remained constant when compared to 0 dpi (Fig 5B). Since the EBOV GP-specific IgG levels remained constant between 0 dpi and 21 dpi, we assessed the antibody binding affinity (Fig 5C). The results indicated an increase in antibody binding affinity of the IN-IN survival group compared to the other exposure groups (Fig 5C). Lastly, neutralizing antibodies were assessed with a recombinant VSV-EBOV-GFP-based assay which demonstrated similar levels between all groups on 0 dpi and only a slight increase in the surviving IN-IN group on 21 dpi (Fig 5D).

### Pilot study: IN-exposed TAFV and EBOV ferrets showed no histopathologic changes

The liver, spleen, and lungs were again assessed for histopathologic changes. Notably, the ferrets meeting endpoint criteria in the IM-IM, IN-IM, and IM-IN groups (n = 2/group) were euthanized 5–7 dpi while the surviving ferrets in the IN-IN group (n = 2) were euthanized at study end (21 dpi).

**Fig 6:**
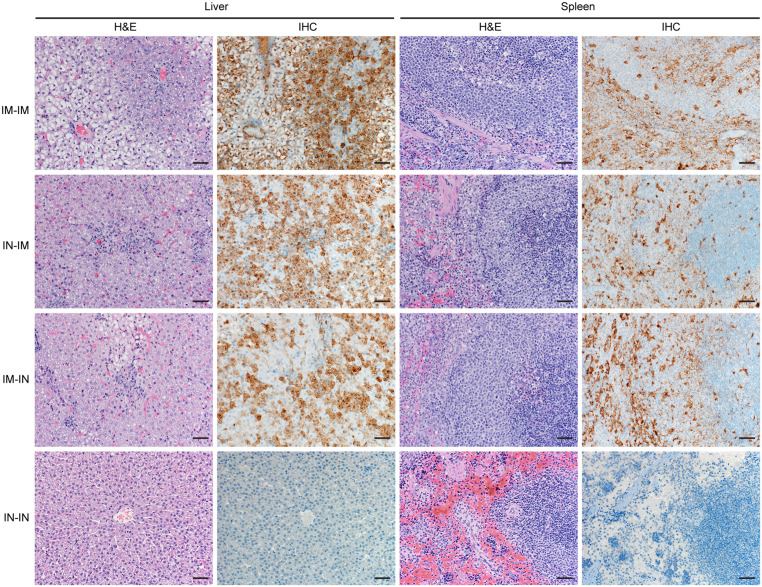
Pathology in EBOV-exposed ferrets. Ferrets were exposed IM or IN to 1,000 TCID_50_ of EBOV after surviving TAFV inoculation. Hematoxylin & eosin (H&E) and immunohistochemistry (IHC) staining in tissues of EBOV-exposed ferrets at 5-7 dpi or at study end (21 dpi). Images 200x magnification. Scale bar represents 50 µm.

Tissue samples from the IN-IN group, which survived both TAFV and EBOV inoculations, showed no evidence of classical filovirus lesions or immunoreactivity in any tissues that were assessed (Figs 6, S7). However, the ferrets in the IM-IM, IN-IM, and IM-IN groups that succumbed to EBOV infection and acute disease demonstrated with classical filovirus histopathology in the liver and spleen. Diffuse immunoreactivity was present in Kupffer cells and hepatocytes as well as splenic macrophages in the red pulp (Fig 6). No pulmonary lesions were evident in the lungs of the ferrets that succumbed, but EBOV-specific immunoreactivity was found in the pulmonary macrophages, endothelial cells, and perivascular alveolar septa (S7 Fig).

## Discussion

TAFV has caused only a single human case of infection which originated from a TAFV disease outbreak in nonhuman primates (NHP) [4], demonstrating that TAFV is a human-pathogenic filovirus that warrants further research. However, small animal disease models for TAFV are currently lacking. Intraperitoneal infection of TAFV in immunodeficient type I interferon receptor knockout (IFNAR^-/-^) mice showed no signs of disease with 100% survival [5]. NHPs are susceptible to TAFV infection and develop disease [5,17,18], however, they are not a feasiblie animal model for initial countermeasure efficacy testing. Ferrets are an established model for mucosal viral infections and are susceptible to wildtype filovirus infection [8,19]. Compared to rodents and NHPs, ferrets are a new model for filovirus inoculation and more research is needed using different exposure routes for human disease comparisons. There is compelling evidence that natural filovirus transmission and infection occurs by mucosal exposure [6,7], therefore, we aimed to develop a small animal disease model using a mucosal route of infection with TAFV.

The IM group had 100% survival while the mucosal infection routes resulted in 83% (IN) and 50% (aerosol) survival. The ferrets developing acute severe disease were euthanized 9–10 dpi presenting with classical filovirus disease demonstrating that ferrets are susceptible to mucosal TAFV inoculation. There were common clinical signs observed regardless of exposure group, such as ruffled fur, hunched posture, and diarrhea. Notably, the ferrets that succumbed to TAFV disease showed signs of labored breathing while the surviving ferrets did not. The most pronounced disease development was observed in the aerosol-exposed ferrets which may be an atypical disease manifestation compared to a natural exposure since the virus was deposited directly into the lungs in a controlled environment and, therefore, requires further research. These ferrets had increased radiograph scores due to lung infiltrates and mild to severe interstitial pneumonia. Interestingly, interstitial pneumonia was previously documented in IM-infected cynomologous macaques with TAFV [5]. However, this was not observed in the current IM-exposed ferrets. The aerosol-exposed ferrets also had increased inflammatory biomarkers representative of classical filovirus disease: IFN-γ, MCP-1, MIP-1β, and IP-10. These biomarkers are produced by filovirus-infected cells and play key roles in the host immune response, such as attracting immune cells to inflammation sites (i.e., monocytes, macrophages, T cells, or NK cells) [20,21].

A study by Schiffman, et. al. showed that TAFV caused mild disease in the ferret model after IM or IN exposure [11]. Both IM exposure outcomes had 100% survival, however, ferrets in the current study developed weight loss, temporary thrombocytopenia, and viremia [11]. The difference was even more notable after IN exposure where they observed minimal disease, but in the current study these ferrets developed thrombocytopenia, neutrophilia, lymphocytopenia, and viremia. Importantly, the current study showed lethal disease in 3/6 ferrets after aerosol TAFV exposure, however, the previous ferret study did not investigate this exposure route [11]. These differences may be due to a 10-fold higher virus dose of 10,000 TCID_50_ to inoculate the current ferrets compared to the previous study which used 1,000 TCID_50_ [11]. Additionally, the viral stocks used for inoculation were different between the two ferret studies which may have contributed to the different outcomes since the potential differences between viral stock propagation may impact pathogenicity in animal models. An example of this is demonstrated in another study where a drastic TAFV disease phenotypic difference was observed in cynomolgus macaques comparing two separate stock preparations containing only seven coding genomic differences between them [5]. The sequence differences are located in genomic regions known to impact viral replication and editing, however, future studies are needed to decipher which changes contribute to the observed phenotypic differences.

Currently, there is limited research on cross-protection between filovirus species and even less with mucosal exposure routes. Ferrets in the current study that survived TAFV inoculation by IM and IN exposures developed IgG responses reactive to both TAFV and EBOV GP. Therefore, cross-protection between these filovirus species was assessed with an EBOV rechallenge pilot study of IM or IN exposure. We divided the TAFV IM- and IN-exposed ferrets into 4 groups, enabling the investigation of previous exposure route impacts on cross-protection to EBOV. Only ferrets exposed IN to TAFV and IN to EBOV were uniformly protected from disease and EBOV viremia. Interestingly, a previous study assessing the protective efficacy of a filovirus vaccine cocktail resulted in two unvaccinated control NHPs surviving IM TAFV infection which where rechallenged IM with either EBOV or SUDV [16]. Both NHPs survived the rechallenge without developing disease and viremia. The EBOV NHP survivor was again rechallenged IM with SUDV and again survived without developing disease or viremia [16]. This is interesting because cross-protection between SUDV and EBOV after IM administration in NHPs has previously not been achieved [14,22] indicating that the TAFV-induced immunity may benefit survival from EBOV or SUDV infection. The current ferret study did not observe cross-protection between TAFV and EBOV after IM exposure, only after IN exposure. These findings highlight the impacts of animal species, exposure routes, and viral stocks used in research models.

The current results could indicate that mucosal immunity after TAFV infection may provide increased protection against exposure to other filoviruses in the ferret model. There is a need to investigate the impact of mucosal immunity (may it stem from a previous infection or a vaccine) on filovirus infection since it is believed to be the natural route of infection of filovirus-exposed survivors. Additional investigation is required in future studies to characterize the potential role of mucosal immunity for the outcome observed in the current cross-protection pilot study. Notably, the impacts of filovirus infection on T cells, epithelial cells, B cells, and dendritic cells is of importance in mucosal immune defense mechanisms and memory/effector functions [23]. These cells play essential roles in maintaining homeostasis, initiation of mucosal immune responses, and induction of secretory IgA which protects mucosal surfaces from the environment [24]. However, little is known about this in the context of mucosal filovirus infection. In the current study, the IN-IN survival group had constant levels of serum binding antibodies, increased antibody affinity, and no tissue damage. Interestingly, antibodies such as secretory IgA can interact with viral antigens and promote neutralization and clearance of the virus without causing tissue damage, which may be contributing to the protection observed in the current study [25].

Our studies contribute to the growing field of filovirus disease research in the ferret model, but there were limitations. These were pilot studies designed to characterize the potential disease and pathogenicity of TAFV in a new animal species model. The limited experimental sample collection did not allow for detailed characterization of cellular phenotyping, transcriptomic analysis, soluble glycoprotein quantification, or viral particle uptake comparisons. Assessment of antibody responses were also limited, including IgM, IgA, and antibody effector functions. Currently, the T cell response and further humoral response analysis in ferrets is also limited due to the lack of available reagents. Other study limitations were related to the specific ferret groups. While the initial TAFV study was performed with mixed sex groups and 6 ferrets per group, the cross-protection study was conducted with only one sex of ferrets per group with small group sizes (n = 2) and should be regarded as a pilot study. It also lacked control groups of TAFV- or EBOV-only inoculations. Aerosol-exposed ferrets were also not assessed due to only 3/6 ferrets surviving TAFV infection which didn’t allow for both accurate comparisons of the pathogenicity study and multiple exposure route comparisons for the rechallenge pilot study. Future studies are needed to confirm our findings and address factors such as sex-bias, nature, and durability of the cross-protective immunity.

Taken together, our data shows that ferrets are a feasible model to assess TAFV pathogenicity by mucosal exposure routes. More importantly, we are the first to demonstrate potential cross-protection between TAFV and EBOV in ferrets after IN infection. These results challenge the existing dogma in the field as cross-protection may be achieved after mucosal infection in ferrets and very likely occurs in the human population since the natural filovirus infection route is mucosal. Further studies will need to investigate the mechanism mediating cross-protection in ferrets and the potential impacts for human disease.

## Materials and methods

### Ethics statement

Filovirus infectious work was performed following standard operating procedures approved by the Rocky Mountain Laboratories (RML) Institutional Biosafety Committee in the maximum containment laboratory at RML, Division of Intramural Research, National Institute of Allergy and Infectious Diseases, National Institutes of Health (NIH). Animal work was performed in accordance with the Guide for the Care and Use of Laboratory Animals of the NIH, the Office of Animal Welfare, and the US Depertment of Agriculture and was approved by the RML Animal Care and Use Committee (ACUC). Procedures were conducted in ferrets anesthetized by trained personnel under the supervision of veterinary staff. Food and water were available *ad libitum*. Endpoint criteria as specified by RML ACUC-approved clinical score parameters were used to determine when ferrets were humanely euthanized.

### Cell line and viruses

Vero E6 cells (*Mycoplasma* negative; ATCC, cat. no. CRL-1586) were grown in Dulbecco’s modified Eagle’s medium (Sigma-Aldrich, St. Louis, MO, USA) containing 4.5 g/L D-glucose supplemented with 10% fetal bovine serum (Wisent Inc., St. Bruno, Canada), 2 mM L-glutamine, 50 U/mL penicillin, and 50 μg/mL streptomycin (all Thermo Fisher Scientific, Waltham, MA, USA) at 37˚C in 5% CO_2_. VSV-TAFV-GFP [5], TAFV and EBOV-Kikwit stocks used were previously described [17,26]. Deep sequencing of all virus stocks revealed no contaminants.

### Ferret study designs

TAFV study: 18 domestic ferrets (*Mustela putorius furo*), 20 weeks old and 0.69–1.52 kg in weight (Triple F Farms, Gillett, PA, USA). On 0 days post-inoculation (dpi), ferrets were inoculated by either IM (0.4 mL/ferret; 2 female/4 male), IN (0.3 mL/ferret; 4 female/2 male), or aerosol (4 female/2 male) routes of exposure with 10,000 TCID_50_ of TAFV [17]. Aerosol inoculation was performed using the AeroMP aerosol management platform (Biaera Technologies, Hagerstown, MD, USA) where aerosol droplet nuclei were generated by a 3-jet collision nebulizer as previously described [27,28]. The study end point was 21 dpi when 6/14 surviving ferrets were humanely euthanized and the remaining 8 ferrets (4 IN, 4 IM) were rested for 36 days before rechallenge with EBOV.

EBOV study: on 0 dpi, 8 ferrets were inoculated by either IM (2 female/2 male) or IN (2 female/2 male) routes of exposure with 1,000 TCID_50_ of EBOV.

For both studies, physical examinations, blood draws, and swab collections were performed on 0, 2, 4, 6, 8, 10, 14, 21 dpi. Body temperature via subcutaneous transponders and body weight were collected daily. All ferrets were observed at least twice daily for clinical signs of disease and humanely euthanized when endpoint criteria were reached.

### Radiographs

Radiographs were obtained as previously described with adaptation to ferrets [29,30]. The following criteria were used to evaluate and apply a score to each image: 0 = normal examination; 1 = mild interstitial pulmonary infiltrates; 2 = moderate interstitial infiltrates (i.e., partial cardiac border effacement, small areas of pulmonary consolidation, alveolar patterns, or air bronchograms); and 3 = pulmonary consolidation as the primary lung pathology, seen as a progression from grade 2 lung pathology.

### Hematology and serum biochemistry

Whole blood (EDTA) samples were collected. Total cell counts were determined via Idexx ProCyte DX analyzer (Idexx Laboratories, Westbrook, ME, USA). Serum biochemistry was analyzed via Vetscan 2 using Preventive care profile disks (Abaxis, Union City, CA, USA).

### RNA extraction and RT-qPCR

Whole blood (EDTA) samples were extracted with QIAmp Viral RNA Mini Kit (Qiagen, Hilden, Germany) and tissues (maximum of 30 mg/tissue) were extracted with RNeasy Mini Kit (Qiagen) according to manufacturer specifications. One-step RT-qPCR was performed with QuantiNova Probe RT-PCR Kit (Qiagen) on the Rotor-Gene Q (Qiagen). Specific TAFV [17] or EBOV [31] primer-probe sets were used as described previously. RNA from the TAFV or EBOV stocks were extracted via QIAmp Viral RNA Mini Kit (Qiagen) and used alongside samples as standards with known concentrations.

### Enzyme-linked immunosorbent assay (ELISA)

Serum was inactivated by γ-irradiation (4 MRad) [32]. ELISAs were performed as previously described [33] using purified TAFV glycoprotein (GP; kindly provided by Dr. Ayato Takada, Hokkaido University, Japan), EBOV GP (IBT Bioservices, Rockville, MD, USA), or EBOV NP (Alpha Diagnostic International, San Antonio, TX, USA) as antigens. The affinity ELISA was performed with previously described EBOV GP antigen [33] at 1 µg/mL. Briefly, antigen was coated at 50 μL/well in a 96-well plate overnight at 4°C. After the antigen was discarded, the plate was blocked with 5% milk at 37°C for 90 min, washed 3 times with PBS/o.o5% Tween-20, and serum incubated in 5% milk at 37°C for 60 min. The serum was discarded, the plate was washed 3 times, and 50 μL/well of ammonium thiocyanate ranging from 4-0.25 M (Millipore Sigma, Burlington, MA, USA) was incubated at 37°C for 15 min. The plate was washed 3 times and secondary antibody in 5% milk (IgG-HRP conjugate; SeraCare, Gaithersburg, MD, USA) was incubated at 37°C for 60 min. HRP substrate was incubated at 21°C for 30 min and adsorption was measured (oD405 nm) on a GloMax (Promega, Madison, WI).

### Serum cytokine and chemokine levels

Irradiated serum samples were assessed via Ferret Cytokine Panel 1 assay (Ampersand biosciences, Lake Clear, NY, USA) according to manufacturer specifications in duplicates. Samples were analyzed on the Bio-Plex 200 system (Bio-Rad, Hercules, CA, USA).

### Virus neutralization assay

Neutralization of irradiated and heat-inactivated serum samples was assessed in Vero E6 cells and performed as previously described [34] with VSV-EBOV-GFP. Samples were analyzed on the FACSymphony A5 Cell Analyzer (BD Biosciences, Mississauga, ON, Canada) where the GFP-positive cell count was measured and data analyzed on FlowJo v.10.

### Histology and immunohistochemistry

Harvested tissues were processed as previously described [17]. Briefly, tissues were fixed in 10% Neutral Buffered Formalin x2 changes, for 7 days minimum. Tissues were placed in cassettes and processed with Sakura VIP-6 Tissue Tek on a 12-hour automated schedule, using a graded series of ethanol, xylene, and PureAffin. Embedded tissues were sectioned at 5 μm and dried overnight (42°C) prior to staining. Immunoreactivity was detected using a previously described cross-reactive rabbit polyclonal anti-EBOV VP40 antibody (1:2000) kindly provided by Dr. Yoshihiro Kawaoka, University of Wisconsin-Madison [35,36]. Secondary antibody was Vector Laboratories ImmPress VR anti-rabbit IgG polymer (# MP-6401). Tissues were stained using the Discovery Ultra automated stainer (Ventana Medical Systems) with a Roche Tissue Diagnostics Discovery purple kit (# 760–229). All tissue slides were evaluated by a board-certified veterinary pathologist. Lung tissue sections (right upper lung lobe, right middle lung lobe, right lower lung lobe, left upper lung lobe, left lower lung lobe, and accessory lung lobe) were evaluated and scored according to the following criteria: 0 = no lesions, 1 = minimal (1–10%), 2 = mild (11–25%), 3 = moderate (26–50%), 4 = marked (51–75%), and 5 = severe (76–100%).

### Statistical analysis

Two-way ANOVA with Tukey’s multiple comparisons was performed between exposure groups for each day. Statistical significance is color-coded to indicate the comparisons in the graphs. Statistics were not performed for the cross-protective study since only n = 2/group was available. Graphs and analyses were performed using GraphPad Prism Software (v. 10.2.0).

## Supporting information

S1 Table Interstitial pneumonia scores after TAFV aerosol exposure in ferrets. (PDF)

S1 Fig Hematology and serological analysis after TAFV exposure in ferrets. (PDF)

S2 Fig Viral load in TAFV-exposed ferrets. (PDF)

S3 Fig Pathology in ferrets that survived TAFV inoculation. (PDF)

S4 Fig Hematology and serum analysis after EBOV exposure in ferrets. (PDF)

S5 Fig Viral load in EBOV-exposed ferrets. (PDF)

S6 Fig Cytokine and chemokine levels in ferret serum after EBOV exposure. (PDF)

S7 Fig Lung histopathology after EBOV exposure. (PDF)
