# Supplementary material for: Mucosal Taï Forest virus infection causes disease in ferrets
Source: PLoS Pathog. 2025 Oct 13;21(10):e1013579. doi: 10.1371/journal.ppat.1013579 (PMC12530580; doi:10.1371/journal.ppat.1013579)
Supplement: S1 Table — (PDF) [file ppat.1013579.s001.pdf]

	Succumbed (10 dpi)			Survived (21 dpi)		
RUL	0	5	2	0	0	1
RML	3	4	4	1	0	0
RLL	4	4	4	0	3	1
LUL	4	3	5	0	2	1
LLL	5	4	5	0	0	2
Acc.	2	4	4	0	0	1

Table S1. Interstitial pneumonia after TAFV aerosol exposure in ferrets. Ferrets were inoculated with 10,000 $TCID_{50}$ of TAFV (n=6/group). The table depicts interstitial pneumonia scores for aerosol-inoculated ferrets only, where n=3 ferrets succumbed (10 days post infection (dpi)) and n=3 ferrets survived (21 dpi). RUL, right upper lung lobe; RML, right middle lung lobe; RLL, right lower lung lobe; LUL, left upper lung lobe; LLL, left lower lung lobe; Acc., accessory lung lobe.
