# Supplementary material for: Mucosal Taï Forest virus infection causes disease in ferrets
Source: PLoS Pathog. 2025 Oct 13;21(10):e1013579. doi: 10.1371/journal.ppat.1013579 (PMC12530580; doi:10.1371/journal.ppat.1013579)
Supplement: S1 Fig — (PDF) [file ppat.1013579.s002.pdf]

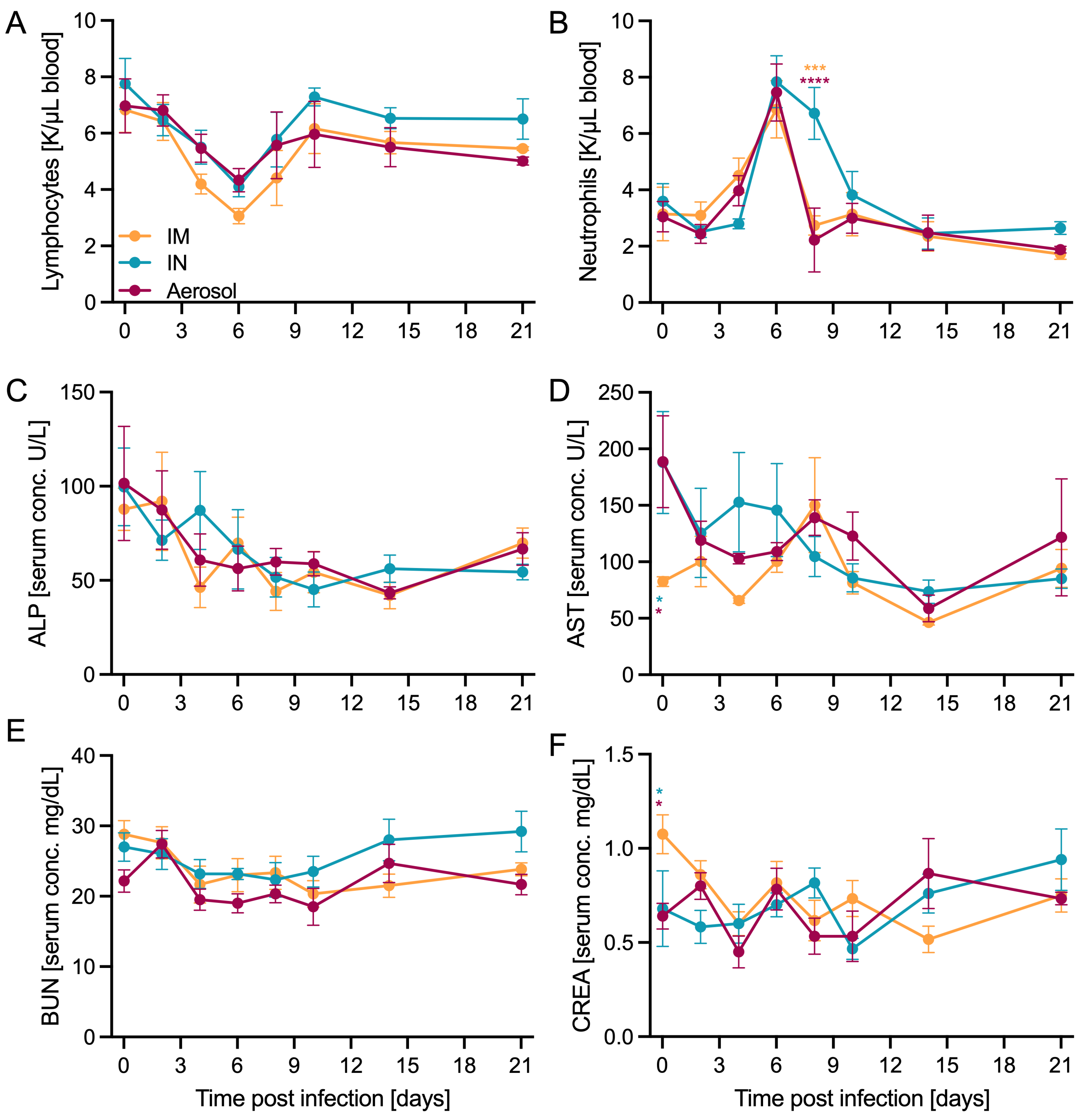

Figure S1. Hematology and serological analysis after TAFV exposure in ferrets. Ferrets were inoculated IM, IN, or by aerosol (n=6/group) with 10,000 $TCID_{50}$ of TAFV. (A) Lymphocyte and (B) neutrophil cell counts in whole blood samples. (C,D) Liver and (E,F) kidney enzyme levels in the serum. All depicted as mean with SEM. ALP, alkaline phosphatase; AST, aspartate transaminase; BUN, blood urea nitrogen; CREA, creatinine. Statistical significance calculated by 2-way ANOVA with Tukey's multiple comparisons is indicated as $^*p<0.05$, $^{***}p<0.001$, and $^{****}p<0.0001$.
