# Supplementary material for: Mucosal Taï Forest virus infection causes disease in ferrets
Source: PLoS Pathog. 2025 Oct 13;21(10):e1013579. doi: 10.1371/journal.ppat.1013579 (PMC12530580; doi:10.1371/journal.ppat.1013579)
Supplement: S2 Fig — (PDF) [file ppat.1013579.s003.pdf]

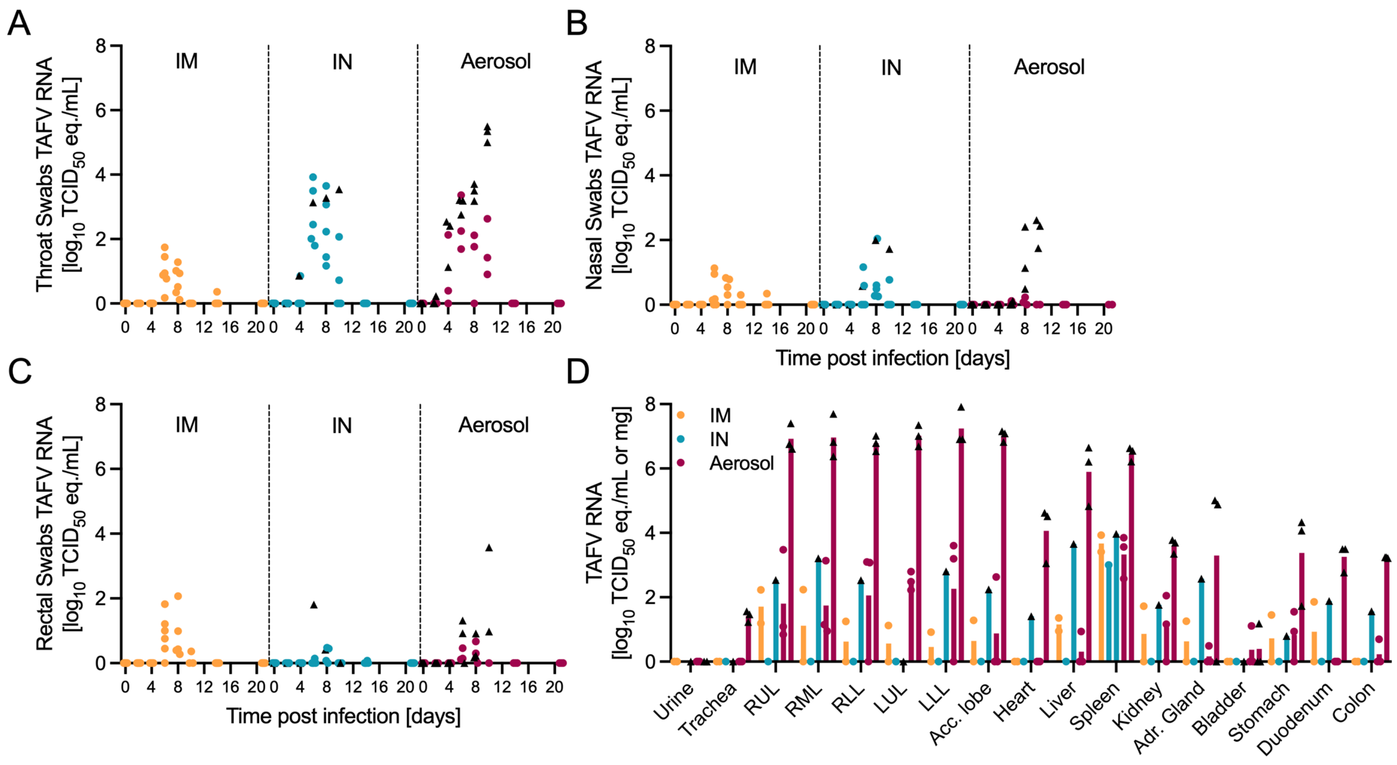

Figure S2. Viral load in TAFV-exposed ferrets. Ferrets were inoculated IM, IN, or by aerosol with 10,000 TCID$_{50}$ of TAFV. TAFV-specific RNA loads in (A) throat, (B) nasal, and (C) rectal swabs throughout the study (n=6/group). (D) Urine and tissue samples from 9-10 dpi or study end (21 dpi). Geometric mean and individual ferrets are depicted. IM n=2; IN n=2; aerosol n=6. RUL, right upper lung lobe; RML, right middle lung lobe; RLL, right lower lung lobe; LUL, left upper lung lobe; LLL, left lower lung lobe; Acc. lobe, accessory lung lobe; Adr. Gland, adrenal gland. ▲Ferrets reaching endpoint criteria.
