# Supplementary material for: Mucosal Taï Forest virus infection causes disease in ferrets
Source: PLoS Pathog. 2025 Oct 13;21(10):e1013579. doi: 10.1371/journal.ppat.1013579 (PMC12530580; doi:10.1371/journal.ppat.1013579)
Supplement: S3 Fig — (PDF) [file ppat.1013579.s004.pdf]

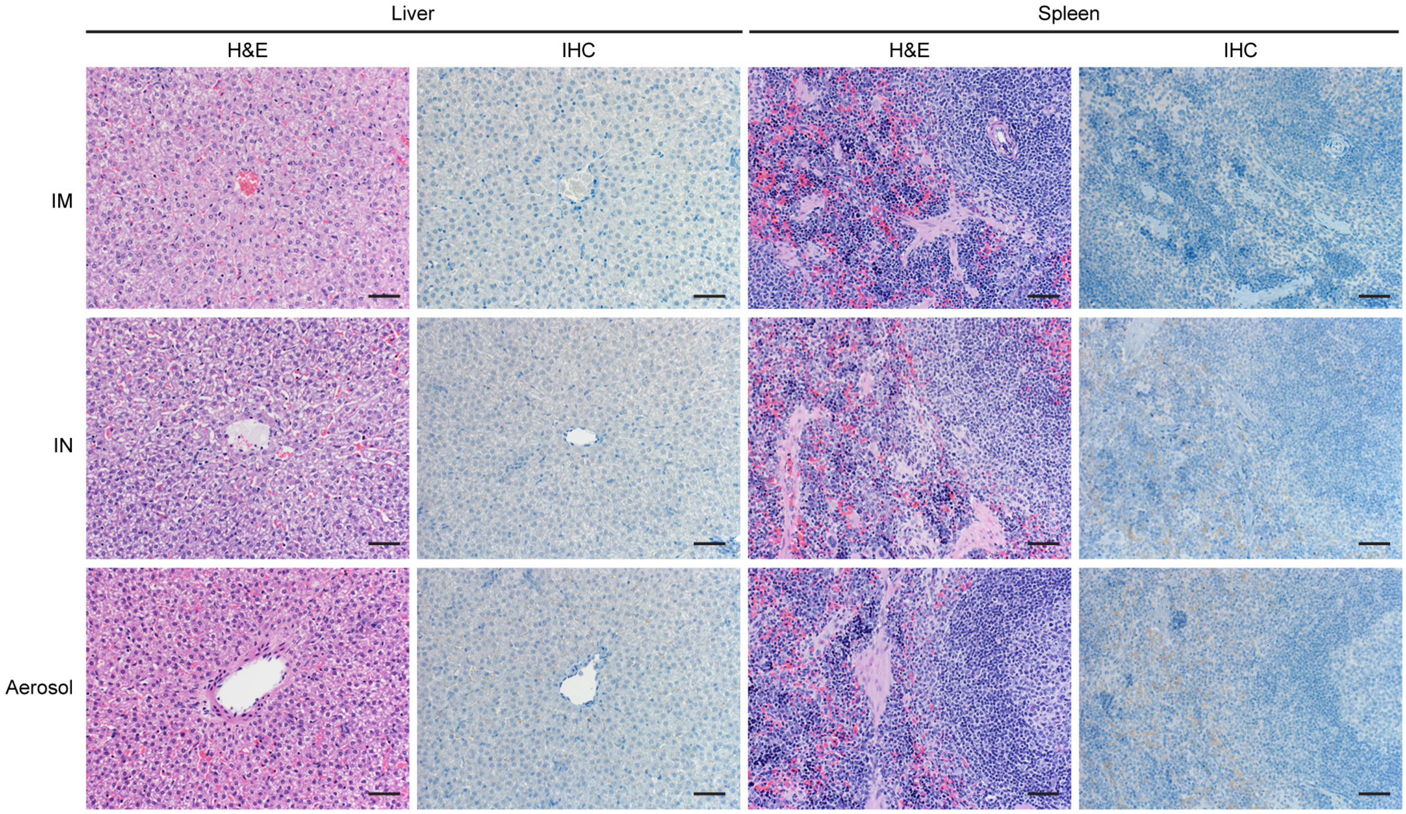

Figure S3. Pathology in ferrets that survived TAFV inoculation. Ferrets were inoculated IM, IN, or by aerosol with 10,000 $TCID_{50}$ of TAFV. Hematoxylin & eosin (H&E) and immunohistochemistry (IHC) staining in tissues of TAFV-exposed ferrets that survived to study end (21 dpi). Images 200x magnification. Scale bar represents 50 µm.
