# Supplementary material for: Mucosal Taï Forest virus infection causes disease in ferrets
Source: PLoS Pathog. 2025 Oct 13;21(10):e1013579. doi: 10.1371/journal.ppat.1013579 (PMC12530580; doi:10.1371/journal.ppat.1013579)
Supplement: S4 Fig — (PDF) [file ppat.1013579.s005.pdf]

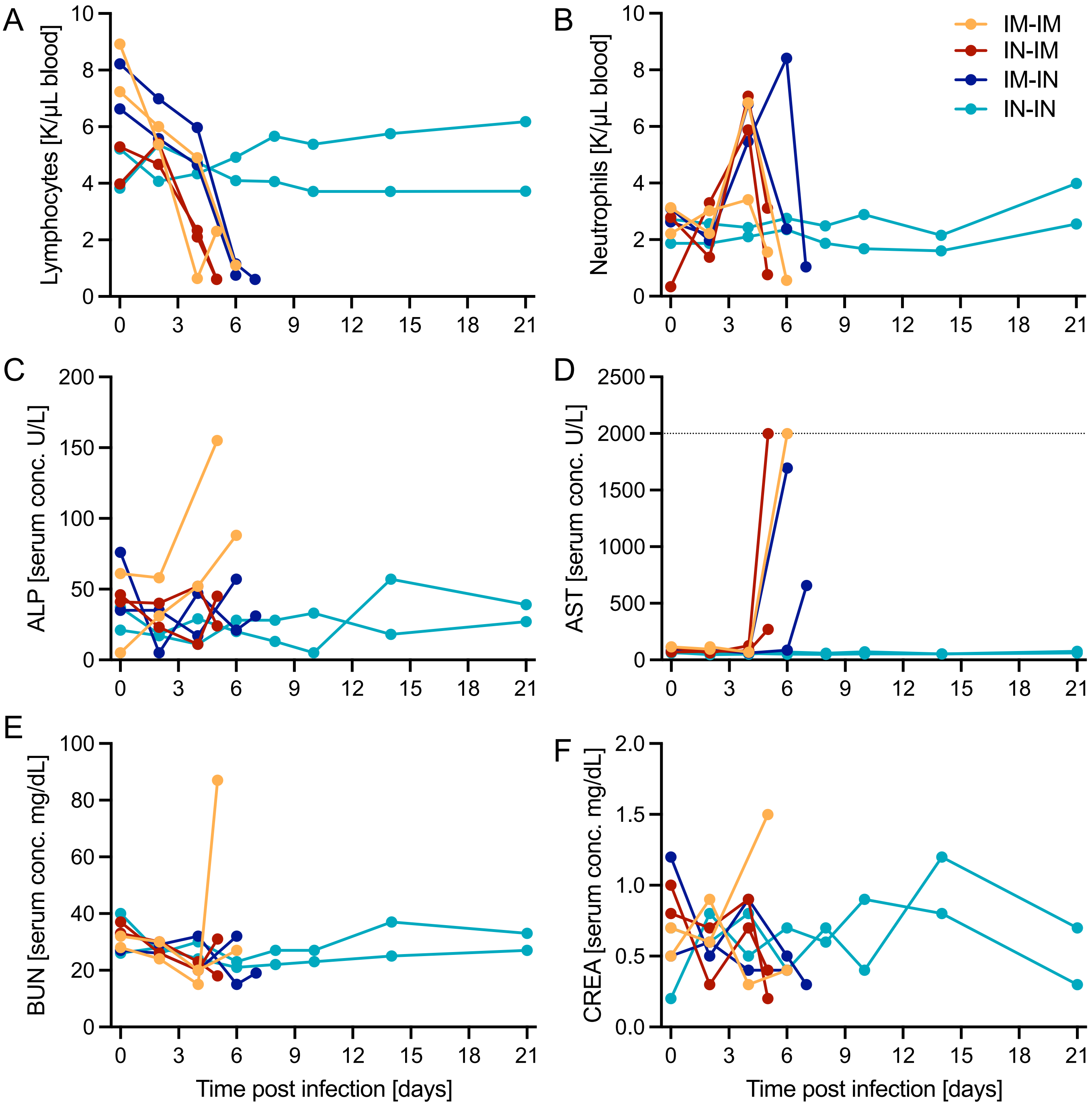

Figure S4. Hematology and serum analysis after EBOV exposure in ferrets. Ferrets were exposed IM or IN to 1,000 $TCID_{50}$ of EBOV (n=2/group) after surviving TAFV inoculation. (A) Lymphocyte and (B) neutrophil cell counts in whole blood. (C,D) Liver and (E,F) kidney enzyme levels in the serum. ALP; alkaline phosphatase, AST; aspartate transaminase, BUN; blood urea nitrogen; CREA; creatinine. Dotted line represents upper limit of detection.
