# Supplementary material for: Mucosal Taï Forest virus infection causes disease in ferrets
Source: PLoS Pathog. 2025 Oct 13;21(10):e1013579. doi: 10.1371/journal.ppat.1013579 (PMC12530580; doi:10.1371/journal.ppat.1013579)
Supplement: S5 Fig — (PDF) [file ppat.1013579.s006.pdf]

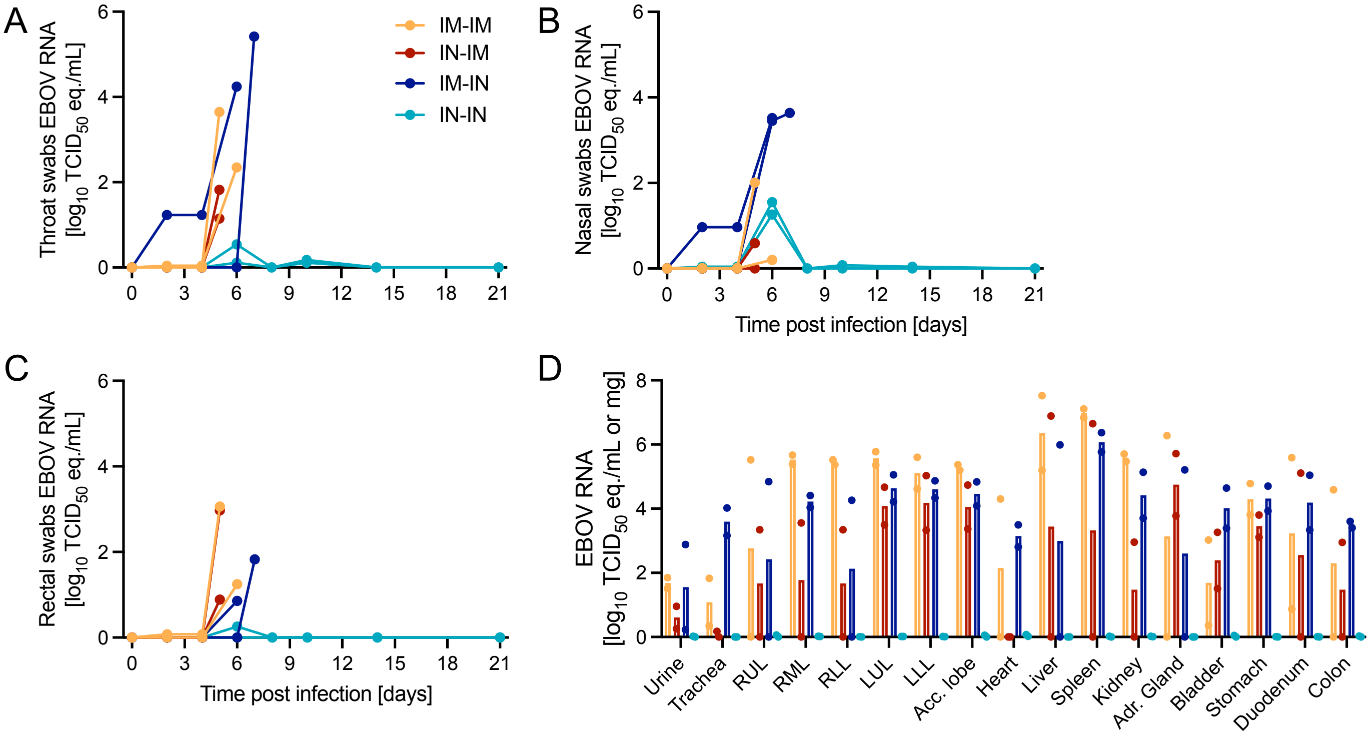

Figure S5. Viral load in EBOV-exposed ferrets. Ferrets were exposed IM or IN to 1,000 $TCID_{50}$ of EBOV (n=2/group) after surviving TAFV inoculation. EBOV-specific RNA loads in (A) throat, (B) nasal, and (C) rectal swabs throughout the study. (D) Urine and tissue samples from 5-7 dpi or study end (21 dpi). Geometric mean and individual data points are depicted. RUL, right upper lung lobe; RML, right middle lung lobe; RLL, right lower lung lobe; LUL, left upper lung lobe; LLL, left lower lung lobe; Acc. lobe, accessory lung lobe; Adr. Gland, adrenal gland.
