# Supplementary material for: Mucosal Taï Forest virus infection causes disease in ferrets
Source: PLoS Pathog. 2025 Oct 13;21(10):e1013579. doi: 10.1371/journal.ppat.1013579 (PMC12530580; doi:10.1371/journal.ppat.1013579)
Supplement: S6 Fig — (PDF) [file ppat.1013579.s007.pdf]

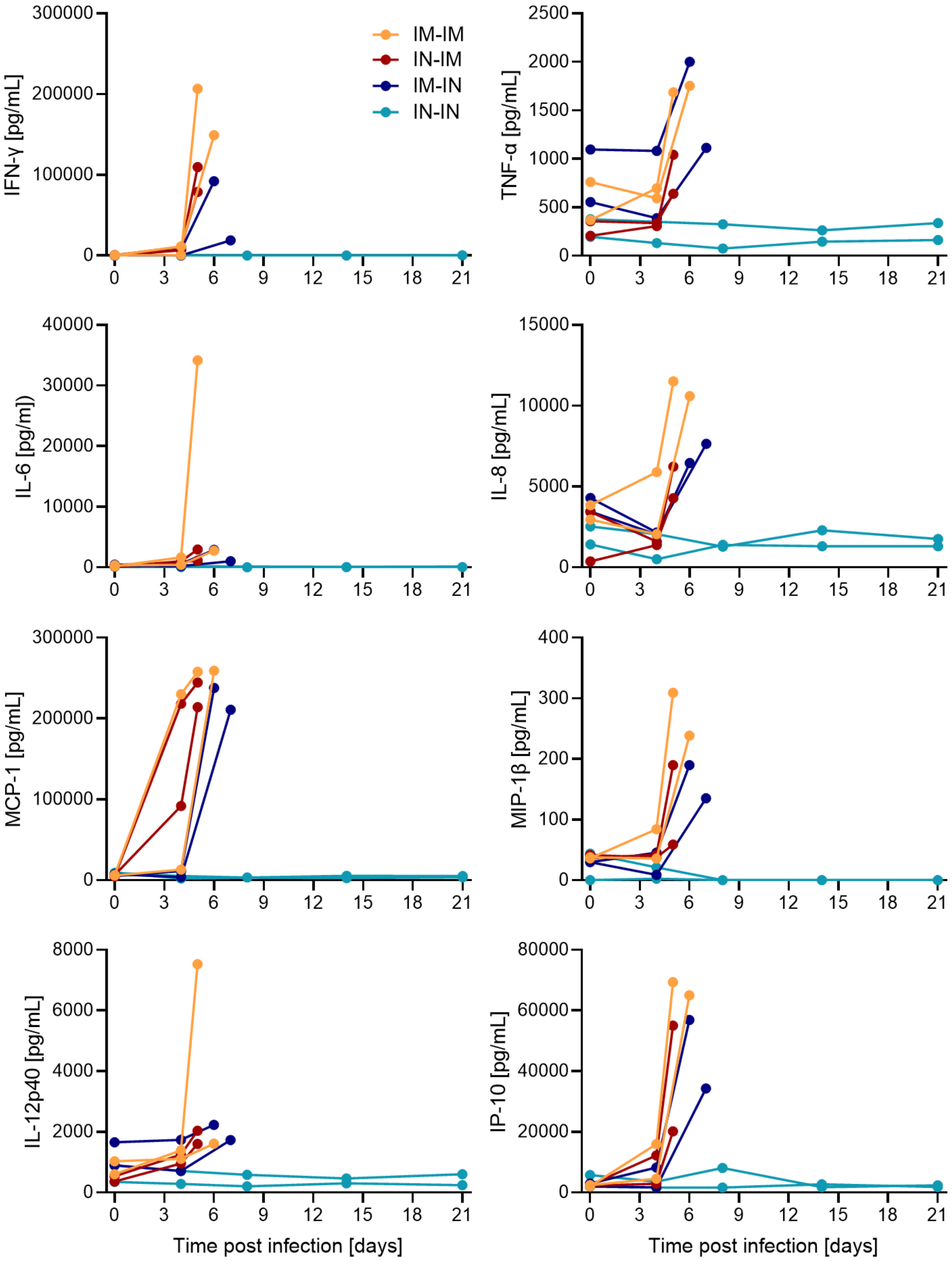

Figure S6. Cytokine and chemokine levels in ferret serum after EBOV exposure. Ferrets were inoculated IM or IN with 1,000 $TCID_{50}$ of EBOV (n=2/group) after surviving TAFV inoculation. Levels of select cytokines and chemokines in serum samples collected from each ferret over time.
