# Supplementary material for: Mucosal Taï Forest virus infection causes disease in ferrets
Source: PLoS Pathog. 2025 Oct 13;21(10):e1013579. doi: 10.1371/journal.ppat.1013579 (PMC12530580; doi:10.1371/journal.ppat.1013579)
Supplement: S7 Fig — (PDF) [file ppat.1013579.s008.pdf]

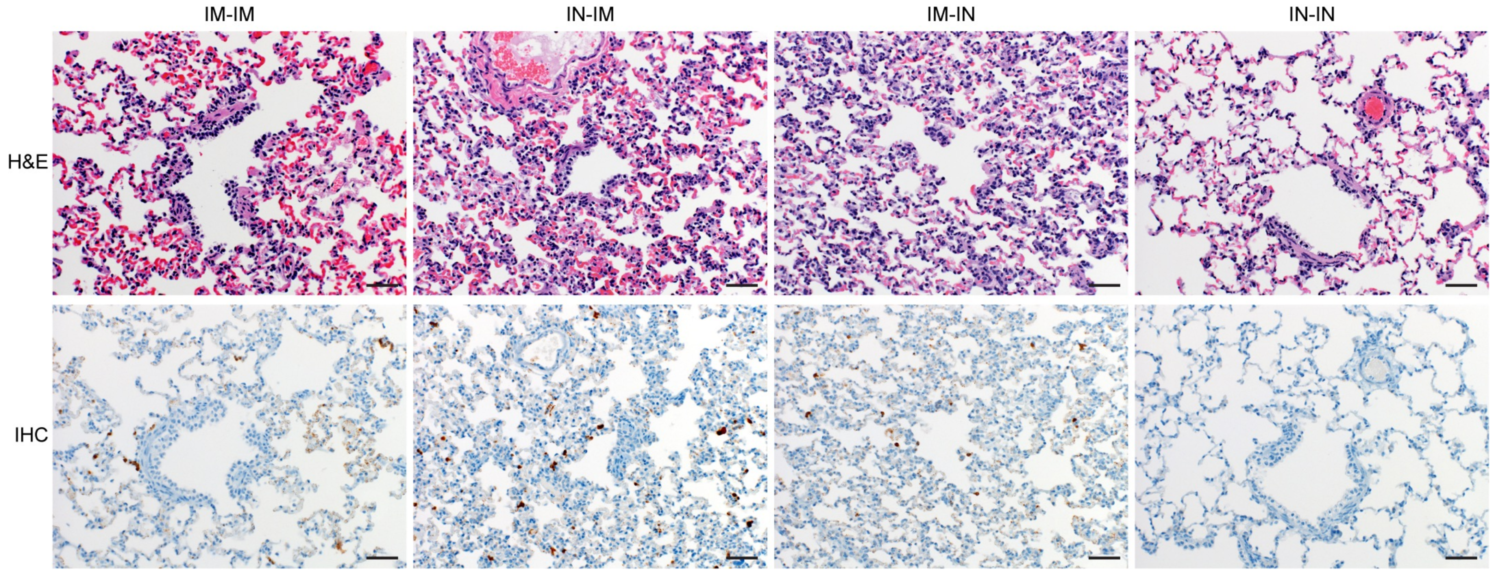

Figure S7. Lung histopathology after EBOV exposure. Ferrets were exposed IM or IN to 1,000 $TCID_{50}$ of EBOV after surviving TAFV inoculation. Hematoxylin & eosin (H&E) and immunohistochemistry (IHC) staining in lung samples of EBOV-exposed ferrets at time of scute disease (5-7 days post infection (dpi)) or at study end (21 dpi). Images 200x magnification. Scale bar represents 50 μm.
